# Impact of peer support on student mental wellbeing: a systematic review

**DOI:** 10.15694/mep.2018.0000170.1

**Published:** 2018-08-14

**Authors:** Nikita Margaret John, Oliver Page, Scott Christopher Martin, Paula Whittaker

**Affiliations:** 1 University of Manchester

**Keywords:** peer support, mental health, wellbeing, university students

## Abstract

This article was migrated. The article was marked as recommended.

**Objectives:** Many universities use peer support programmes to support students. However, there are currently no guidelines on the most effective way of facilitating emotional wellbeing in students. The aim of this paper is to review the evidence for the effectiveness of peer support to improve mental health wellbeing in university students.

**Methods:** Six electronic databases (Medline, Embase, British Education Index (BEI), Australian Education Index (AEI), PsychINFO and Applied Social Sciences Index and Abstracts (ASSIA)) were searched in December 2017. Search terms included “peer support”, “university students” and “mental wellbeing”. Studies that did not include peer support or assess the impact of students’ mental health were excluded. Data about design and delivery of the peer support intervention and changes in student mental wellbeing outcomes were extracted.

**Results:** 489 records were identified. Three studies met the inclusion criteria; two cross-sectional surveys and one non-randomised intervention study. One study found social support to be the most important protective factor for mental wellbeing. Two studies showed no statistically significant improvement in wellbeing outcomes with peer support. One study found that majority of mentors and mentees found peer support useful.

**Conclusions:** There is currently no evidence that peer support improves mental wellbeing among university students.

## Introduction

Student lifestyles in different universities around the world can vary massively ( Yu Y et al, 2015). The academic and social pressures endured also vary among individuals. Individuals react differently to circumstances because resilience levels vary among people ( Waaktaar and Torgersen, 2012). University is a time of transition both academically and socially associated with significant lifestyle changes, and students inevitably adapt to these in different ways ( Maunder, 2013).

A large UK study found very high rates of self-reported clinical anxiety in male (23%) and female (35%) students ( Andrews and Wilding, 2010). There is also a general trend of increased examinations, heightened aspirations, financial pressures and increasingly impersonal institutions that are faced by university students ( Royal College of Psychiatrists, 2003). The increased external pressure to competitively succeed in academic goals adds to self-imposed expectations, which may lead to unhealthy stress levels in students ( El Ansari et al, 2013). The undergraduate university period also demands increasing adult responsibility but also allows increased freedom for lifestyle choices. First year students experience higher stress levels than those in later years ( Ramsay, Jones, and Barker, 2007) suggesting that students struggle, especially initially, with the social adjustments. For example, a study found that students in their first term at university gained an average of 1.3-3.1kg, and were more susceptible to starting smoking and/or excessive alcohol consumption ( El Ansari et al, 2013) highlighting how vulnerable university students are to developing unhealthy habits that may lead to poor physical and psychological wellbeing. Despite these pressures being well documented, there is limited research into the relationship between adjustment and support processes during this period of transition ( Ramsay, Jones, and Barker, 2007).

University students have poorer mental health than similar aged individuals in the general population ( Andrews and Wilding, 2010). Studies from Sweden, Germany and the UK also show that students appear to have a lower perceived quality of life than that of same-age young people in the general population ( El Ansari et al, 2013). Although university students complain more about their health than their working counterparts, most do not seek help for these problems ( El Ansari et al, 2013). Despite this growing concern surrounding mental health in students, the effectiveness of student counselling has been a neglected research area ( Connell, Barkham, and Mellor-Clark, 2008).

Support can be delivered in various ways. For example: emotional (communication to make others feel valued), practical (material assistance), informational (passing knowledge through guidance and advice), and social companionship (spending time in leisure activities) ( Cohen and Wills, 1985).

Currently most universities employ counselling services, but they often focus on students who already have a pre-diagnosed mental health issue ( Connell, Barkham, and Mellor-Clark, 2008). One survey of 978 students studying at a university in Australia found that 27.6% agreed that they needed help (for issues related to education, future prospects and social/personal issues). Of those who felt they needed help, just 19.8% actually visited a counselling service. The reasons for this were because they felt the problem was not sufficiently important, they felt uncomfortable asking for help or they felt the service provider would not understand them ( Russell, Thomson, and Rosenthal, 2008).

Students also get support from their social network of family and friends through face-to-face and social media connections. These methods of support have been shown to reduce feelings of depression ( Wright et al, 2013). People who report higher levels perceived social support, also report lower levels of all types of loneliness ( Bernardon et al, 2011). For university students living away from home, social support from peers may be more important than from family members ( Bernardon et al, 2011).

Peer support is about offering understanding and care to someone empathetically through sharing emotional and psychological experiences, and is a system of giving and receiving help respectfully in a mutual agreement ( Mead, Hilton and Curtis, 2001). Mead, Hilton and Curtis (2001) propose that this is easier to practice with peers than others, such as professional counsellors, because people find affiliation with individuals they feel are similar to them, and as trust in the relationship builds, both people are able to respectfully challenge each other, which allow members of the peer community to reflect on their behaviours. Research indicates that perceiving one’s environment as supportive can reduce the psychological impact of stressful events ( Ramsay, Jones, and Barker, 2007). Furthermore, students tended to feel personally validated when they received support from others, which contributed to positive coping at university (Terenzini et al, 2011). This led to improved outcomes for students, including in academic achievement ( Ramsay, Jones, and Barker, 2007).

## Aim

The aim of this systematic review is to evaluate the effectiveness of peer support in improving mental health wellbeing outcomes for university students.

## Methods

### Literature search and data sources

On December 28 2017, six electronic databases: MEDLINE, EMBASE, PsychINFO, ASSIA, BEI and AEI were systematically searched. Key words used for the search strategy were “peer support”, “university student” and terms relating to mental wellbeing such as “loneliness” and “stress”. A base search was constructed on MEDLINE, and then translated onto other databases. The search strategy was kept as identical as possible; however, MeSH terms had to be adapted for different databases (see appendix I). Duplicates were removed using Endnote reference manager and manually. The remaining unique articles were subjected to title and abstract screening independently by three authors (NJ, OP and PW). The remaining full texts were reviewed independently by three authors (NJ, OP and PW). Discrepancies were resolved by consensus discussion chaired by the third author (PW). Data from the included studies was extracted independently by three authors (NJ, SM and OP). Relevant data included study design, participant demographics, intervention method, how wellbeing was measured and the outcomes.

### Eligibility criteria

The inclusion and exclusion criteria focused on removing papers that did not quantitatively measure indicators of mental health wellbeing in university students. Articles must have included university students with no or only mild-to-moderate mental health diagnosis, any intervention that incorporated peer support, and have measured indicators of wellbeing quantitatively. Articles must have been written in English and have been available as a full-text version. Conference abstracts were not included, but efforts were made to identify all published work based on their content, including grey literature. Articles that were delivered in a group and facilitated by a “non-peer” were included if there was evidence of active peer support occurring within the group.

### Quality assessment

The quality of the included studies was assessed independently by three authors (NJ, SM and PW). The risk of bias assessment for the included studies was completed using the Risk Of Bias In Non-randomised Studies- of Interventions tool (ROBINS-I).

## Results

**Figure 1.**
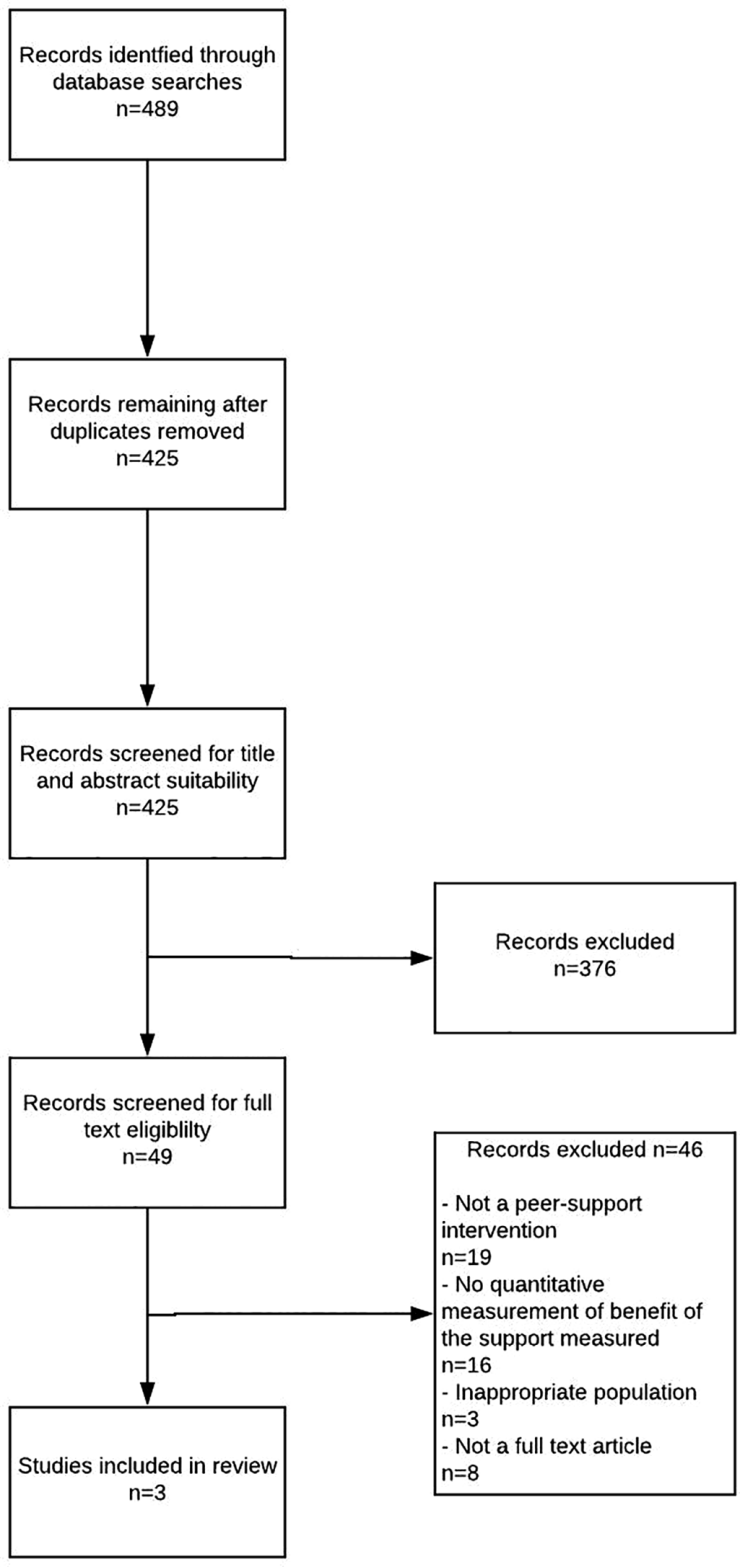
PRISMA flowchart of literature review process

Searching the six databases yielded 489 citations; after deduplication, 425 titles and abstracts were reviewed. 49 papers were eligible for full text review. Three full text articles met the inclusion criteria. The reasons for exclusion of the 46 papers excluded in the last stage of screening are: not a peer support intervention (n=19), no qualitative measurement of benefit of the support measured (n=16), inappropriate population (n=3), not full-text article (n=8), (see figure 1).

### Peer support design

All three included studies provided different types of peer support. Twomey (1991) compared one-to-one support with group support. Loane (2015) measured the effectiveness of signposting to supportive interventions through a peer support intervention for mentors and mentees. Biro, Veres-Balajti, and Kosa (2016) measured students’ general self-reported “perceived peer support” within their current programme. A summary of the included is found in table 1 in the supplementary material.

### Student selection

All studies recruited students through voluntary participation. Biro, Veres-Balajti, and Kosa (2016) included 130 participants who were physiotherapy students studying in years one to three at a University in Hungary. Loane (2015) included 178 participants that were all first year students from the University of Western Sydney. Twomey (1991) included 41 participants who were first year students (or students who had been out of studies for several semesters) that studied psychology and sociology at New Mexico State University.

### Measurement of peer support outcomes

All three studies measured outcomes using questionnaire surveys. Biro, Veres-Balajti, and Kosa (2016) used a questionnaire survey that incorporated various scales, including: Mean score for Sense of coherence Scale (SoC), social support from peers (non-validated questionnaire), notable psychological distress (General Health Questionnaire), and perceived stress (Perceived Stress scale). The variables of mental health (SoC, social support from peers, notable psychological distress and perceived stress) were compared among different sexes of students.

Loane (2015) used a questionnaire survey that asked non-validated questions answered on a Likert scale. The questions asked were to assess the desired outcomes of the peer support training and intervention for the peer mentors and mentees respectively.

Twomey (1991) used a pre- and post- peer support intervention questionnaire to compare changes in positive affect (The Bradburn (1969) Affect Balance measure), negative affect (The Bradburn (1969) Affect Balance measure) and quality of life (The Andres and Whithey (1976) Quality of Life measure).

### Main findings

Biro, Veres-Balajti, and Kosa (2016) found that 68% of female students and just 38% of male students studying physiotherapy reported feeling full support from their peers (p=0.045), and students reported a 1.5 fold reduction in peer support from year 1 (70%) to year 3 (53%). Support from peers was found to increase SoC scores but the result did not reach statistical significance (p=0.092).

Loane (2015) found that the majority of mentors and mentees found the peer support programme to be beneficial in helping to connect with student support services in university.

Twomey (1991) found no statistically significant changes in negative affect or quality of life following the peer support intervention. Positive affect decreased in mentees in the “one-to-one” peer support comparison group (from 4.16 out of 5 to 2.56), whereas it increased in the “group” comparison group (from 3.83 to 3.92). However, the largest increase in affect was seen in the control group (from 3.72 to 4.6).

### Quality of included studies

Biro, Veres-Balajti, and Kosa (2016) and Twomey (1991) were assessed by ROBINS-1 to have a low risk of bias whist Loane (2015) was assessed to have a high risk of bias. Twomey (1991) identified age as a confounding variable and adjusted the results to account for this. There was moderate bias due to missing data due to some drop-outs from the intervention groups. However, the outcome measurements were done consistently across groups and all outcomes measured were reported in the results. Both Twomey (1991) and Biro, Veres-Balajti, and Kosa (2016) contained more female than male participants. Biro, Veres-Balajti, and Kosa (2016) analysed the results for males and females separately to control for this potential confounding factor.

Biro, Veres-Balajti, and Kosa (2016) used validated tools to measure wellbeing outcomes and all outcomes measured were reported. Loane (2015) did not report results for all the questionnaire items that they collected, indicating possible selection bias. Furthermore, the response rate of the survey was very low and outcome measurements were subjective. Although the overall this study was assessed at being at high risk of bias, the risks of confounding variables and bias in participation selection and bias in classification of interventions were low. Domains Biro, Veres-Balajti, and Kosa (2016) Twomey (1991) Loane (2015) 1 Low Low Low 2 Low Moderate Low 3 Low Low Low 4 N/A Moderate Moderate 5 Low Moderate High 6 Low Low High 7 Low Low High Overall Low Low High

**Table 2.** Summary of risk of bias for 3 reviewed articles

Domains	Biro, Veres-Balajti, and Kosa (2016)	Twomey (1991)	Loane (2015)
1	Low	Low	Low
2	Low	Moderate	Low
3	Low	Low	Low
4	N/A	Moderate	Moderate
5	Low	Moderate	High
6	Low	Low	High
7	Low	Low	High
Overall	Low	Low	High

## Discussion

### Key findings

This review has not found evidence to suggest that peer support programmes increase the mental wellbeing of university students. Biro, Veres-Balajti, and Kosa (2016) report that although females felt more supported by their peers, they had worse self-reported wellbeing. Interestingly, Twomey (1991) found that peer support did not cause significant changes in negative affect or quality of life measures, but one-to-one support actually was detrimental to positive affect. These two articles indicate that peer support does not facilitate improved mental wellbeing. In contrast, Loane (2015) reported that a peer support intervention was useful in connecting students to student service that could help them in crisis. However, Loane (2015) did not report evidence to indicate the effectiveness of the peer support delivered by the mentors to the mentees.

### Behaviour in Females versus Males

Two of the three included articles reported a higher proportion of participation from females ( Biro, Veres-Balajti, and Kosa, 2016) ( Twomey, 1991). This may be partly explained by the increasing proportion of females entering higher education ( Hillman and Robinson, 2015). However, participants in both studies were almost exclusively female ( Biro, Veres-Balajti, and Kosa, 2016) ( Twomey, 1991). This was perhaps because participation to engage in the peer support programme was voluntary with no other incentives, and females are more likely to associate with similar others ( Szell and Thurner, 2013), and engage with help-seeking behaviours better than men ( Thompson et al, 2016). Twomey (1991) agrees with the concept that “we like people and situations which provide reinforcement for us”, however does not state that this theory is limited for females.

Twomey (1991) matched all mentors and mentees by similar characteristics of age and sex. Gender was a covariate they considered but the comparison groups did not have significantly different numbers, as there was only one male in the individual group and one male in the larger group. However, in the control group there were five males, and positive affect in the control group had improved the most between measured time periods. This imbalance between the genders in the intervention groups and control group may explain why the control group had better outcomes than the intervention groups ( Twomey, 1991).

Self-reported outcomes may be misleading because women are less hesitant to express emotion and weakness ( El Ansari et al, 2013). Furthermore, males seek help much less frequently than their female counterparts, which may explain why men are four times more likely to commit suicide ( Värnik, 2012). Mental ill health is very common in men, with 10-15% of men experiencing a major depressive episode in their lifetimes, yet generally they are less willing to acknowledge their weaknesses or get help compared to women ( Wendt and Shafer, 2016).

### Different agendas for peer support

The delivery and purpose of peer support offered to students varies between universities. Some institutions have peer mentors trained specifically to deliver services, whereas other universities take a less structured approach. Loane (2015) described a peer support intervention designed to promote students’ sense of belonging and boost their retention through skills in motivation, listening, empathy and building trust. Twomey (1991) described the delivery of structured peer support through one-to-one or group sessions to allow new students to interact with older and successful students who can provide a positive example and be encouraging by normalizing similar stresses they encountered. Programmes that use peer mentors as an instructional resource are linked to a more supportive campus culture ( Colvin and Ashman, 2010) however it is not clear whether they improve mental wellbeing.

The University of Manchester’s Medical School runs an internal peer support network, known as the ‘Mummies and Daddies’ scheme ( University of Manchester, 2016). This scheme offers social support to students in their first year of medical school and first year of clinical studies. This mentoring is delivered by peer mentors in the year above. Their roles are to offer support through social activities, and they are not trained ‘instructional resources’ ( University of Manchester, 2016).

Many peer mentor programmes focus on new students, to integrate them into university life. Studies have recommended stress management particularly for first years ( Park et al, 2015). Evidence has shown that first year students experience higher levels of stress than those in later years, and that they are more likely to struggle with social adjustments ( Ramsay, Jones, and Barker, 2007), suggesting that students may need peer support more at the beginning of their university life and less as they progress. However, studies comparing peer support across different years of study show that the more senior the student, the less supported they feel by their peers ( Biro, Veres-Balajti, and Kosa, 2016) ( Park et al, 2015).

Byrom (2017) compared 23 peer programmes run to tackle depression among students. It was not included in this review because the participants had pre-existing severe mental health diagnoses. However, it is worth noting that participation in the peer support programmes was associated with a decrease self-reported depression scores, and the author assessed peer support as being equally as useful as professional counselling and significantly better than no treatment ( Byrom, 2017). This is in contrast to the finding of Twomey (1991) who found that peer support produced no significant difference in negative affect scores. The discrepancy could be due to differences in the peer support delivered and differences in the outcome measures used. A study comparing peer support delivered via face-to-face interaction versus social media interaction indicates that some methods may be more effective than others ( Wright et al, 2013). It is therefore important to collect data comparing different methods of delivering peer support to ensure the optimised methods are employed by universities to improve mental wellbeing in their student populations.

Other studies have looked at social support from family members and other networks that may be able to support students, such as their lecturers ( Biro, Veres-Balajti, and Kosa, 2016) ( Basson and Rothmann, 2018). Basson and Rothmann (2018) recognise the importance of peer support as students have daily contact with their peers and can meet each other’s psychological needs by acknowledging feelings. They found that support from peers and family played a significant role in satisfaction levels reported by students, and that family was considered the most important source of support ( Basson and Rothmann, 2018). Bernardon et al (2011) disagree, and argue that support from peers is more important than from family, because university students have more interactions and similar experiences to their peers than their family.

It is difficult to judge how popular the uptake of peer support is among students. Two of the three articles included in this review include data from voluntary surveys ( Biro, Veres-Balajti, and Kosa, 2016) ( Loane, 2015). Twomey (1991) was the only study that reported a planned intervention, but the participation was voluntary and the sample size small. Byrom (2017) used a larger sample size and delivered their programme across eight different UK universities. Only 34% of participants completed the intervention programme, but 69% of those who completed the peer support intervention felt the session had improved their ability to look after their own mental health ( Byrom, 2017). Thus, the intervention had a significant impact on mental wellbeing for those who engaged with it. Engagement in peer support is consistently higher in women, because are more likely to ask for help ( Thompson et al, 2016). So, although peer support programmes may encourage students to seek support earlier, peer support intervention designs should be mindful that the methodology retains student engagement and appeals to both sexes.

### Limitations of the review

The key limitation of this review is that only three articles were included. It is possible that relevant articles were not identified during the search. The methodology excluded qualitative studies which may have provided valuable insights into this topic. Furthermore, the quality of the included studies was not robust. The review may have been affected by language bias, as only articles available in English were included.

### Implications for practice and research

Peer support methods vary in delivery and agenda. A degree of peer support is practiced in most universities, ranging from formally trained mentors whereas to informal social contact. Although peer support methods have been adopted in many universities worldwide, details about the delivery methods and the impact on the participants are not well documented. Even when attempts are made to evaluate the peer support programme, the measurement of the outcomes are not adequately recorded.

## Conclusion

This review is inconclusive in answering whether peer support among university students is effective in improving mental wellbeing. It is recommended that universities should document their peer support activities and measure their results to provide an evidence-base for effective interventions.

## Take Home Messages

• Peer support schemes are widely used in universities
• There are currently no guidelines on the most effective way of facilitating emotional wellbeing in students
• There is currently no evidence that peer support improves mental wellbeing among university students
• Universities should evaluate the effectiveness of their peer support activities and share the findings to build an evidence-base for effective interventions

## Notes On Contributors

Nikita Margaret John is a fifth-year medical student at The University of Manchester. She has an interest in Health economics and is involved with integrating more mindfulness and empathy in medical education. She has a first-class bachelor’s degree in Management and Innovation in Healthcare from the University of Manchester.

Paula J. Whittaker is a Senior Clinical Lecturer in Public Health at the University of Manchester and Honorary Consultant in Public Health England. She is the programme director of the BSc Management and Innovation in Healthcare and deputy programme director of the MPH.

Oliver Page is a medical student at The University of Liverpool. He has a special interest in Healthcare Management and Dermatology. He has a first-class bachelor’s degree in Management and Innovation in Healthcare from the University of Manchester.

Scott C. Martin is a fifth-year medical student who has started his own enterprise ‘Ai Patient’. He has a first-class bachelor’s degree in Management and Innovation in Healthcare from the University of Manchester.
