# Supplementary material for: Impact of peer support on student mental wellbeing: a systematic review
Source: MedEdPublish (2016). 2018 Aug 14;7:170. [Version 1] doi: 10.15694/mep.2018.0000170.1 (PMC10701817; doi:10.15694/mep.2018.0000170.1)
Supplement: Peer review history

# Reviewer report: Reviewer response for version 1

**DOI:** 10.21956/mep.19621.r29184

**Published:** 2020-01-08

**Authors:** Stephanie Traudt

**Affiliations:** 1 Central Michigan University

This review has been migrated. The reviewer awarded 4 stars out of 5 Nice article--would just like to point out that in the paper itself Terenzini et al. (2011) is cited, but the references include a Terenzini et al. (1994) citation.

Reviewer Expertise:

NA

No decision status is available

# Reviewer report: Reviewer response for version 1

**DOI:** 10.21956/mep.19621.r29182

**Published:** 2019-04-30

**Authors:** Saee Deshpande

**Affiliations:** 1 Maharashtra University of Health Sciences, Nashik- VSPM Dental College Nagpur

This review has been migrated. The reviewer awarded 4 stars out of 5 very elaborate review on an important topic.would like to know why just single date and not time frame of articles published is mentioned?as mentioned in previous review, qualitative studies could have been included too.however, overall a well written article indeed!

Reviewer Expertise:

NA

No decision status is available

# Reviewer report: Reviewer response for version 1

**DOI:** 10.21956/mep.19621.r29185

**Published:** 2018-08-17

**Authors:** Stefanie Hautz

**Affiliations:** 1 I

This review has been migrated. The reviewer awarded 4 stars out of 5 Dear authors, thank you very much for adding your article to the discussion on peer support regarding wellbeing of students. The article is of interest for all planning to develop and/or implement such a program. The review is well written and performed very thoroughly. Thus, I would like to add a few suggestions.As for the methods, is there a reason why you did not include PubMed as a data base for your search of articles? If so, please indicate. Also, it would be beneficial to the reader if you could briefly state why one exclusion criteria was to imit to quantitative measures only (why no qualitative?). Please note that there is a misleading spelling mistake between your text and Figure 1 (once it is labeled as “qualitative measurement of benefit of the support measured” and once as “quantitative measurement of...”).As for the results, I suggest to include the measurement outcomes of Loane 2015 within the text also, not only in your appendix. Also, is there a percentage or quote for the results “furthermore, the response rate of the survey was very low (how low?) and outcome measurements were subjective (example?)”. Last, I suggest to cite ROBINS-1 (e.g.: Sterne Jonathan AC, Hernán Miguel A, Reeves Barnaby C, Savović Jelena, Berkman Nancy D, Viswanathan Meera et al. ROBINS-I: a tool for assessing risk of bias in non-randomised studies of interventions BMJ 2016; 355 :i4919 ) and also name the domains in your table rather then to put the numbers 1-7 there, as you did in your appendix. If would be of more comfort to the reader.Again, thank you for your great work and the very successful article.

Reviewer Expertise:

NA

No decision status is available

# Reviewer report: Reviewer response for version 1

**DOI:** 10.21956/mep.19621.r29183

**Published:** 2018-08-14

**Authors:** Trevor Gibbs

**Affiliations:** 1 AMEE

This review has been migrated. The reviewer awarded 4 stars out of 5 A very worthwhile paper to read and one that, for a change, openly discusses the negative as much as the positive. Papers such as this are important, especially the review type, in order that we do not accept what is proposed to be a given and can propose that this given is not based upon fact.As the authors rightly point out, a potential weakness is the lack of papers from their search, but given the inclusion parameters, once must respect their findings. An interesting paper for all those involved in student welfare.

Reviewer Expertise:

NA

No decision status is available
